# Impact of patient-reported outcomes on symptom monitoring during treatment with checkpoint inhibitors: health-related quality of life among melanoma patients in a randomized controlled trial

**DOI:** 10.1186/s41687-022-00414-5

**Published:** 2022-01-21

**Authors:** Lærke K. Tolstrup, Helle Pappot, Lars Bastholt, Sören Möller, Karin B. Dieperink

**Affiliations:** 1 Department of Oncology, Odense University Hospital, Odense, Denmark; 2 Department of Clinical Research, University of Southern Denmark, Odense, Denmark; 3 Department of Oncology, Copenhagen University Hospital, Copenhagen, Denmark; 4 OPEN – Open Patient Data Explorative Network, Odense University Hospital, Odense, Denmark

**Keywords:** Melanoma, Immunotherapy, Immune checkpoint inhibitors, Adverse events, irAEs, HRQoL, QoL, Fact-M, EQ-5d-5L, Patient reported outcomes, ePRO, PRO

## Abstract

**Introduction:**

In a randomized controlled trial, we previously investigated if melanoma patients receiving checkpoint inhibitors had fewer severe immune-related adverse events (irAEs) when they reported symptoms using electronic patient-reported outcomes (ePRO) with triggered alerts as an add-on to standard care, compared to standard care alone. The aim of this study is to examine between-group differences in health-related quality of life (HRQoL) and associations between irAEs severity and HRQoL.

**Methods:**

The study population of 138 patients completed the EuroQol EQ-5D-5L Index and FACT-M questionnaires at baseline and weeks 24 and 48. We analyzed HRQoL from all patients who completed at least one questionnaire. Missing FACT-M items were imputed following existing guidelines.

**Results:**

There was no difference in HRQoL at baseline as measured EQ-5D-5L between the intervention and the control group. Between baseline and 48 weeks, mean EQ-5D-5L scores were unchanged among patients in the intervention group (*p* = 0.81) but decreased significantly among patients in the control group (*p* = 0.03). Consequently, patients in the intervention group had higher mean scores than those in the control group (*p* = 0.05) at 48 weeks. Mean FACT-M scores did not differ significantly between the two groups at any of the time points. There were observed no between-group differences in mean EQ-5D-5 and mean FACT-M scores between patients with severe irAEs and patients who had none.

**Conclusion:**

Melanoma patients receiving CPIs who self-reported irAEs using ePRO with triggered alerts as a supplement to standard care maintained their HRQoL compared to patients who received standard care alone. Patients in the intervention group had a significantly better HRQoL measured by EQ-5D-5L than controls 48 weeks after baseline. The results suggest that including ePRO in standard care increases melanoma patients´ well-being. Further and larger studies are needed to confirm this finding and examine the impact of severe irAEs on cancer patients’ HRQoL.

*Trial registration*: Clinicaltrials.gov *NCT03073031 Registered 8 March 2017, Retrospectively registered* https://clinicaltrials.gov/.

**Supplementary Information:**

The online version contains supplementary material available at 10.1186/s41687-022-00414-5.

## Background

Since the introduction of ipilimumab (anti-CTLA-4) in 2011, other checkpoint inhibitors (CPIs) have been approved alone or in combination for the treatment of malignant melanoma. This has led to significant improvements in survival [1]. More than half of melanoma patients who receive combination therapy with Ipilimumab and Nivolumab (anti-PD-1) are alive after 5 years [2]. However, several randomized controlled trials (RCTs) within the last decade have also demonstrated that patients may experience severe immune-related adverse events (irAEs) such as diarrhea, skin toxicity, neurological problems, or endocrine disorders during treatment [3–5]. These irAEs may be life threatening or long term. Patients who receive combination therapy can be particularly affected, with more than half of those receiving combination CPIs experiencing grade 3 or 4 irAEs [2]. The importance of early detection of irAEs is repeatedly stressed [6]. If detected in time, appropriate prompt treatment can be initiated, preventing irAEs from becoming severe [4]. One way to optimize early detection may be to include patients in reporting symptoms. In a RCT (PROMelanoma), we previously investigated whether melanoma patients who received CPIs would have the number of severe irAEs (grade 3 or 4, according to the Common Terminology Criteria for Adverse Events [7]) reduced by collecting electronic patient-reported outcome (ePRO) data [8]. We did not find this to be the case. Patients in the intervention (ePRO with triggered alerts and clinician feedback during clinical encounters as an add-on to standard care) and control (standard care) groups experienced similar numbers of irAEs. However, patients in the intervention group telephoned the hospital significantly more often, suggesting increased attention to side effects [8].

We did not only use ePRO to detect irAEs. As a secondary endpoint, we collected health-related quality of life (HRQoL) data. Other studies have shown that patient-reported outcome data (PRO) and the derived benefits positively affect cancer patients´ HRQoL. In this context, HRQOL covers the subjective perceptions of the positive and negative aspects of cancer patients’ symptoms, including physical, emotional, social, and cognitive functions and, importantly, disease symptoms and side effects of treatment [9]. Basch et al. [10] found that patients receiving chemotherapy for mixed cancer diagnoses who reported symptoms between visits had improved HRQoL, compared with patients receiving standard care. Similarly, in a recent RCT, Absolom et al. reported that cancer patients receiving adjuvant chemotherapy who participated in online symptom reporting experienced improved physical well-being and self-efficacy, compared with usual care [11]. However, to the best of our knowledge, no study has examined the impact of using PROs during treatment with CPIs.

Some severe irAEs patients experience during treatment with CPIs reverse quickly, while others persist long term. Some may even become irreversible [12]. Independent of their length and severity, irAEs may affect patients detrimentally, substantially decreasing HRQoL [13]. However, other studies indicate that melanoma patients treated with CPIs maintain better HRQoL than those treated with other anti-neoplastic therapies [12, 14] and, aside from fatigue, long-term melanoma survivors have moderate symptom burden and good quality of life (QoL) one year after treatment initiation [15]. The fact that patients experience severe irAEs may not automatically result in impaired HRQoL. A study published in 2018 suggests that cumulative toxicity scores comprising all-grade adverse events (AEs) better reflect impact on QoL than high-grade AEs alone [16]. In addition, patients who experience moderate or even mild irAEs may experience substantial HRQoL impacts because adverse events are long term or particularly troublesome, occur regularly, interfere with daily activities [17], or lead to psychological distress [18, 19].

This study had two objectives. First, we examined between-group differences in HRQoL. We hypothesized that patients assigned to the ePRO intervention would have better QoL than those in the control group (standard care). Secondly, we examined associations between irAE severity and HRQoL.

## Materials and methods

### Setting and patients

Patients were eligible for the study if they had been diagnosed with metastatic melanoma and scheduled to receive CPI(s) at the Department of Oncology at Odense University Hospital in January 2017-May 2019 and agreed to participate in the PROMelanoma RCT. Trial participants randomized to the intervention group completed weekly electronic questionnaires about toxicity and paper-based HRQoL questionnaires at baseline and 24 and 48 weeks. All participants provided verbal and written consent, and the study was registered at the Danish Data Protection Agency (19/41148). According to Danish law, approval from an Ethics Committee was not required.

### RCT design

Patients were randomly assigned and allocated sequentially numbered containers in a 1:1 ratio using the software program Open Patient Explorative Network [20] to either the intervention or control group. Patients in the control group received standard care, while patients in the intervention group received standard care and also reported irAEs electronically from home once a week for 24 weeks. A questionnaire was specifically designed for the patient population using the patient-reported outcomes version of the Common Terminology Criteria for Adverse Events (PRO-CTCAE) platform [21]. Patients in the intervention group received a tablet computer, and introduction to the system and baseline registration were made at the clinic. The software platform AmbuFlex [22] was used. The patients were asked to report their symptoms on a fixed weekday, making reporting easier to remember. When the patients reported a mild or higher irAE, an alert was triggered for the majority of irAEs telling the patient to contact the hospital in case of the emergence of a new symptom or worsening of an existing one. The patients were instructed to contact the usual nurses´ line like other patients. The alert was triggered for 24 out of the 29 items included in the questionnaire. Five symptoms were not at risk of becoming severe overnight. Furthermore, the patient reporting was used actively during the clinical encounter to optimize communication, making it possible to focus on the symptoms that the patients found most burdensome [8]. In addition to the RCT, a mixed methods study was also carried out evaluating patient and clinician experience with the intervention [23].

### HRQoL assessments

In addition to completing the PRO-CTCAE questionnaire, trial participants completed two HRQoL instruments, the generic EuroQol EQ-5D Index and the general FACT-M questionnaire. The patient reporting was used passively as the responses were not applied in the clinical trajectory. The EQ-5D-5L questionnaire has demonstrated face and content validity [24] and has also proved valid with regard to selected measurement properties [25]. It assesses five dimensions (mobility, self-care, usual activities, pain/discomfort, and anxiety) on a five-point Likert scale (no problems, slight problems, moderate problems, severe problems, and extreme problems).

Responses to each dimension are converted into a five-digit index describing the respondent’s health state. Index values were derived from a crosswalk index value calculator developed for Danish respondents, ranging from 0 (dead) to 1 (full health) [26].

The FACT-M questionnaire has also demonstrated face and content validity, which has been confirmed by psychometric testing [27]. It was selected because it assesses the unique concerns of melanoma patients. The FACT-M total score (range, 0–172) was used, which is the sum of scores on four general subscales (physical, social, emotional, and functional well-being) and the melanoma subscale. A total of 43 items were scored from 0 to 4 to represent the degree of frequency or difficulty with each, with higher scores representing better HRQoL. The independent melanoma surgery subscale (MSS) was excluded because it was not relevant for all patients.

Patients were asked to complete both HRQoL questionnaires at baseline and at weeks 24 and 48 whether they were still receiving treatment or had stopped treatment due to disease progression or unacceptable toxicity.

### Statistical considerations

Categorical baseline characteristics were reported as counts and proportions and compared between groups by the chi-squared test, and continuous characteristics were reported as medians with range and compared by the median k-sample test for equal medians. We included HRQoL data from all patients who had completed at least one questionnaire at baseline, week 24, or week 48. Data were not imputed for the EQ-5D-5L questionnaire. If a patient failed to respond to one of the five questions, the questionnaire was discarded. Missing responses on the FACT-M were imputed following FACIT scoring guidelines [27], which was done by multiplying the sum of the subscale by the number of items in the subscale, then dividing by the number of items actually answered [28].

Changes in HRQoL scores from baseline to 24 and 48 weeks were analyzed with linear mixed-effects models that included a fixed-effects interaction between treatment group and time point (baseline, 24, 48 weeks) and a random intercept for each patient. Stata 16 (College Station, TX) was used for all analyses [29].

## Results

### Baseline characteristics

Participants in the intervention and control groups had similar baseline characteristics (Table 1). The median age in both groups was 66 years (range, 32–87), and 53% (78) of participants in both groups were male. Two-thirds (101, 69%) of all patients had ECOG performance status 0, and a similar proportion (98, 67%) received pembrolizumab or nivolumab as monotherapy. Twenty-four (16%) patients in both groups who received adjuvant therapy were all treated with nivolumab. Only seven (5%) patients received ipilimumab. Less than one third (41, 28%) received combination therapy with iIpilimumab and nivolumab.

**Table 1:** Participant baseline characteristics

	Control	Intervention
	N = 73 (%)	N = 73 (%)
*Age*		
Median (range)	66 (32–83)	66 (34–87)
*Sex*		
Male	43 (59)	35 (48)
Female	30 (41)	38 (52)
*Drugs received*		
Ipilimumab	3 (4)	4 (6)
Pembrolizumab	36 (49)	38 (52)
Nivolumab	13 (18)	11 (15)
Ipilimumab + nivolumab	21 (29)	20 (28)
*ECOG performance*		
0	52 (72)	49 (69)
1	19 (26)	19 (27)
2	30 (41)	38 (52)
*Disease stage*		
Stage III	12 (16)	10 (14)
Stage IV	61 (84)	63 (86)
*Line of therapy*		
Adjuvant	13 (18)	11 (15)
1st line	52 (71)	52 (71)
2nd line	6 (8)	6 (8)
3rd line	2 (3)	4 (5)

### ePRO intervention

The majority of patients (52, 78%) complied with the ePRO intervention on a weekly basis, either throughout the whole period (n = 31) or until disease progression or intolerable toxicity (n = 21). The average number of reporting was 17 weeks. A minority of patients (15, 22%) reported more sporadically [8]. There was no difference between the two groups in the number of severe irAEs (*p* = 0.98). However, as a result of responding to the questionnaires and the triggered alerts, the number of phone contacts was significantly higher in the intervention group (*p* = 0.01). Similarly, patients in the intervention group had more extra visits (including emergency room visits), but it was not statistically significant [8]. A majority of patients expressed that they believed that alerts were triggered quite frequently, indicating that the threshold for triggers was too low [23]. Overall, patients reported that they were satisfied with the intervention, and that the increased focus on side effects made them feel more involved in treatment and care [23].

### Response rate for HRQoL questionnaires

After excluding eight patients who experienced disease progression or death within weeks of randomization (n = 6) or withdrew their consent to participate (n = 2) in PROMelanoma, the study population consisted of 138 patients (control group, 71; intervention group, 67). Ninety-four percent (130) participants responded to the baseline questionnaires; due to an error, four patients in each group did not receive the questionnaires. At week 24, approximately 76% (104) patients completed the questionnaires. The remaining 26 patients had died or were terminally ill (14%, 18), did not complete the questionnaires (10%, 13), or it was not administered to them in error (2%, 5). At week 48, the response rate had declined to 54% (75) because 23% (30) of initial participants had died and 26% (34) did not respond for the reasons described above (Table 2). The response rate at week 48 was higher in the intervention group than in the control group, but the difference was not statistically significant (*p* = 0.38).

**Table 2:** Completion rates over time for HRQoL questionnaires

	FACT-M		EQ-5D-5L	
	Intervention group	Control group	Intervention group	Control group
	n (%)	n (%)	n (%)	n (%)
Baseline	63 (94)	67 (94)	63 (94)	67 (94)
Week 24	51 (76)	53 (75)	52 (78)	54 (76)
Week 48	38 (56)	35 (49)	39 (58)	36 (51)

#### EQ-5D-5L

**Fig. 1:**
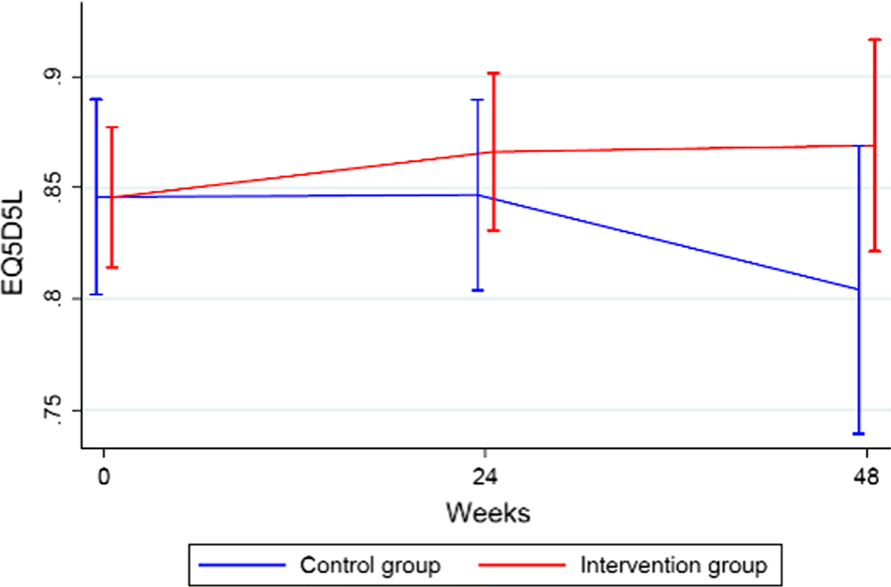
EQ-5D-5L data for patients in the PROMelanoma study at baseline, week 24 and week 48, comparing the HRQoL between patients assigned to the ePRO intervention and patients who received standard care

Baseline EQ-5D-5L QoL scores did not differ between groups. For patients in the intervention group, there was no change in mean score between baseline and week 48 (0.85 and 0.87, *p* = 0.81). However, the mean EQ-5D-5L score declined among patients in the control group from baseline to week 48 (0.85 and 0.80, p = 0.03). There was no between-group difference in mean EQ-5D-5L scores at week 24 (Fig. 1), but the mean score in the intervention group was significantly higher in the control group at week 48 (*p* = 0.05).

**Fig. 2:**
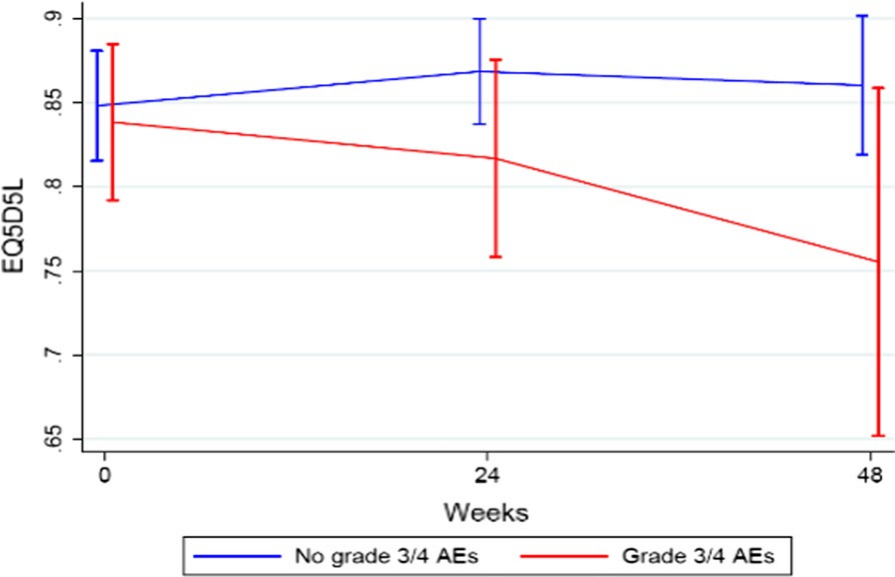
EQ-5D-5L data for patients in the PROMelanoma study at baseline, week 24 and week 48, comparing the HRQoL between patients with no severe irAEs and patients with severe irAEs

Although mean HRQoL scores at week 24 and at week 48 were lower among patients who had experienced severe (grade 3 or 4) irAEs than among patients who had experienced none, mild, or moderate irAEs, (Fig. 2), it did not reach statistical difference (*p* = 0.17 at week 24, *p* = 0.15 at week 48). Additional file 1: Tables S1 and S2 contain more detailed EQ-5D-5L results.

#### FACT-M

**Fig. 3:**
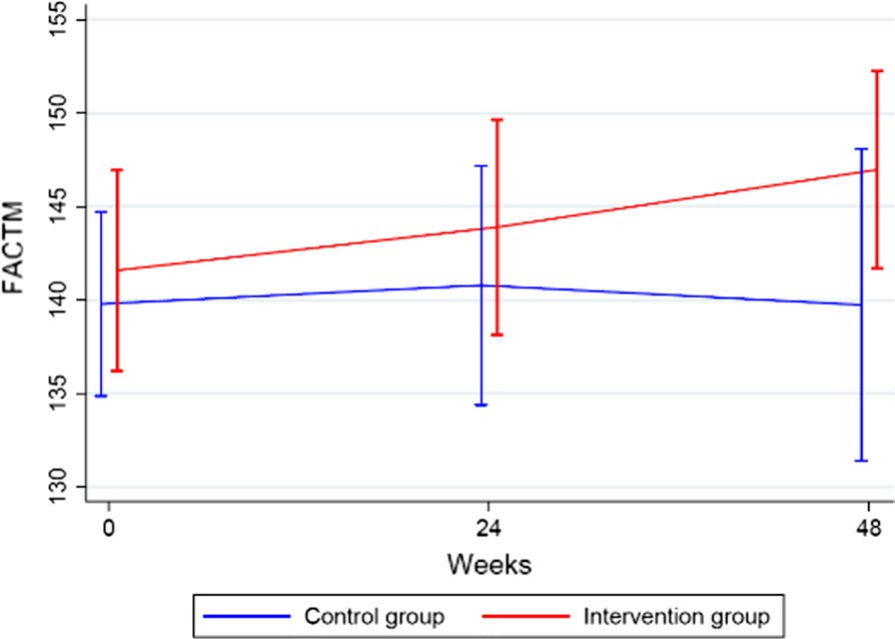
FACT-M data for patients in the PROMelanoma study at baseline, week 24 and week 48, comparing the HRQoL between patients assigned to the ePRO intervention and patients who received standard care

Mean FACT-M scores did not differ between groups (intervention group, 142) and (control group, 140) at 24 weeks (*p* = 0.80). At week 48, the mean FACT-M score in the intervention group was a little bit higher than in the control group (147 and 140, Fig. 3). Although the mean score increased somewhat for patients in the intervention group and remained unchanged for patients in the control group, the difference was not statistically significantly (*p* = 0.12).

**Fig. 4:**
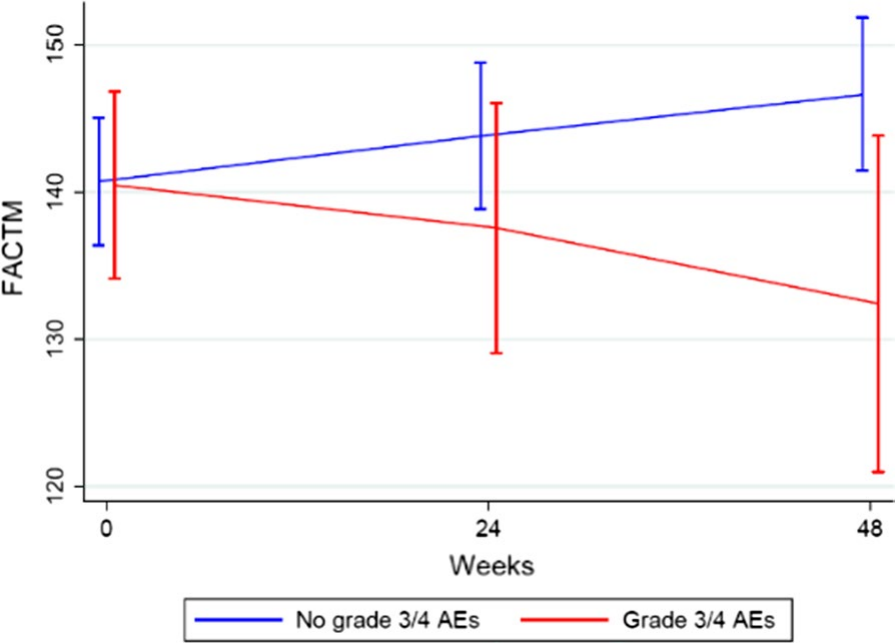
FACT-M data for patients in the PROMelanoma study at baseline, week 24 and week 48, comparing the HRQoL between patients with no severe irAEs and patients with severe irAEs

Mean HRQoL scores at week 24 and at week 48 were lower among patients who had experienced severe (grade 3 or 4) irAEs than among patients who had experienced no severe irAEs, (Fig. 4). However, it again did not reach statistical significance (*p* = 0.28 at week 24, *p* = 0.18 at week 48). Additional file 1: Tables S1 and S2 contain more detailed FACT-M results.

## Discussion

Our findings suggest that melanoma patients receiving CPIs who self-reported irAEs using a tailored questionnaire with triggered alerts as a supplement to standard care have better HRQoL as measured by the EQ-5D-5L than patients receiving standard care.

Patients who were randomized to the intervention group in the PROMelanoma study called the department significantly more often than patients in the control group did and also had more clinic visits [8], potentially due to the alerts in the PRO interface. If they had been inclined to contact the hospital for irAEs or other concerns, doing so may have become more legitimate and reassuring during the RCT. Patients in the intervention group may have felt that their problems were taken seriously and addressed, resulting in a sense of reassurance and maintained HRQoL. Similarly, other studies have found that patients participating in a PRO intervention have increased HRQoL [10, 11] and patient satisfaction [30]. LeBlanc et al. argue that incorporating ePRO data into standard health care settings seems to improve the quality of care for cancer patients [31]. In alignment with these findings, in the mixed methods study among the same patient population, the vast majority of patients assigned to the PRO intervention were extremely satisfied with it [23]. They felt that it was reassuring to be part of the intervention and that they were more involved in their treatment and care. A 2019 review addressing QoL in melanoma survivors also suggests that improving patients´ subjective wellbeing may potentially reduce the emotional and physical consequences of metastatic disease [12]. Thus, the derived benefits from using PROs may increase patients´ wellbeing. In this connection, it is important to keep in mind that if the commonly suggested value of 0.5 standard deviations is used as indicating a clinically relevant change [32], we observed that neither the changes in the EQ-5D-5L score, nor the changes in FACT-M are large enough to be considered clinically relevant.

The gap in HRQoL between the two groups widened over time, even though patients only reported irAEs in PRO-Melanoma for 24 weeks. This may indicate that the increased focus on reacting on irAEs may have a long-lasting effect and that the impact on HRQoL may be even greater over the long term. However, our limited sample size may have precluded detecting statistical significance for even a large difference.

As mentioned in the introduction, discussion continues about the extent to which having severe irAEs impacts patients´ HRQoL. A 2019 study among patients with lung cancer concluded that those who experienced negative feelings about side effects had worse HRQoL than those with positive feelings about side effects [18]. It may be the case that patients who experience irAEs that are severe enough to result in a significant impact on daily life or hospitalization may have negative feelings about AEs, resulting in lower HRQoL compared to patients who experience mild or no AEs. Conversely, studies also indicate that patients who experience irAEs also have a better treatment response [33]. A 2020 study also concluded that the presence of irAEs may be a potential predictive indicator for treatment response and overall survival [34]. In both studies, mild irAEs also predicted a better treatment response. Some patients are aware of these findings, which may cause them to view their irAEs as something positive regardless of severity. Nevertheless, our study indicated that severe irAEs (grade 3 or 4) may tend to negatively affect HRQoL, although this trend did not reach statistical significance.

Study strengths include the use of an RCT to evaluate the secondary endpoint of HRQoL. Patients were stratified by treatment regimen because HRQoL is likely to differ based on the CPI(s) administered [33]. In addition, the questionnaire to monitor symptoms was specifically designed for melanoma patients receiving CPI(s) [21], precisely assessing the toxicities they may experience as recommended by Kluetz et al. [35]. Shojima et al. [36] also argue that AEs vary across treatment regimens, making it difficult to evaluate them. In our study, all patients had melanoma and were treated with drugs with the same toxicity profile, substantial strengths in studying AEs among patients with cancer.

An obvious limitation is the relatively small sample size, particularly at week 48. Some non-respondents were able to complete the questionnaires at this time point but chose not to. If HRQoL had been the primary study endpoint, we may have carried out a more rigorous data collection, by sending out reminders, for example. In this way, we may have obtained a higher number of completed questionnaires [37].

As described in the introduction, previous studies have reported that PRO interventions improved cancer patients´ HRQoL. However, many of these studies had a more complex intervention than ours. For example, we could have included a self-management feature, containing guidance on what to do in case of symptoms. This approach would have been more proactive and empowering for patients [11, 38]. Moreover, patient reporting could have been monitored on a routine basis by a physician to ensure that patients were contacted in case of alerts [10, 11]. In our study [8], patients determined how to respond when an alert was triggered. However, despite our relatively simple intervention, HRQoL was higher in the intervention group; had we included a more proactive intervention, the between-group difference may have been larger.

## Conclusion

Patients with malignant melanoma treated with CPI(s) who self-reported irAEs using PRO with triggered alerts as a supplement to standard care maintained their HRQoL, compared with patients receiving standard care alone. Patients in the intervention group had a significantly better HRQoL as measured by EQ-5D-5L than patients in the control group 48 weeks after baseline. The results suggest that including ePRO in standard care increases melanoma patients´ well-being. Further and larger studies are needed to confirm this finding and examine the impact of severe irAEs on cancer patients’ HRQoL.

## Supplementary Information

**Additional file 1. Supplemental table 1:** Overview QoL data (FACT-M and EQ-5D-5L) for melanoma patients included in the PROMelanoma study - - intervention group vs. control group. **Supplemental table 2**: Overview QoL data (FACT-M and EQ-5D-5L) for melanoma patients included in the PROMelanoma study - grade 3 or 4 irAEs vs. no grade 3 or 4 irAEs

## Data Availability

Informed consent forms, QoL questionnaires, and patient data are stored at the Department of Oncology, Odense University Hospital, Denmark.
