# Supplementary material for: Impact of patient-reported outcomes on symptom monitoring during treatment with checkpoint inhibitors: health-related quality of life among melanoma patients in a randomized controlled trial
Source: J Patient Rep Outcomes. 2022 Jan 21;6:8. doi: 10.1186/s41687-022-00414-5 (PMC8782960; doi:10.1186/s41687-022-00414-5)
Supplement: Supplementary file 1 — Additional file 1. Supplemental table 1: Overview QoL data (FACT-M and EQ-5D-5L) for melanoma patients included in the PROMelanoma study - - intervention group vs. control group. Supplemental table 2: Overview QoL data (FACT-M and EQ-5D-5L) for melanoma patients included in the PROMelanoma study - grade 3 or 4 irAEs vs. no grade 3 or 4 irAEs [file 41687_2022_414_MOESM1_ESM.docx]

|  | FACT-M | | EQ-5D-5L | |
| --- | --- | --- | --- | --- |
|  | **Intervention** | **Control** | **Intervention** | **Control** |
|  | Coefficient (95% CI) P-value | Coefficient (95% CI) P-value | Coefficient (95% CI) P-value | Coefficient (95% CI) P-value |
| Change baseline to 24 weeks | -0.20 (-4.61; 4.21) P=0.929 | 1.52 (-2.74; 5.79) P=0.484 | 0.00 (-0.04; 0.04) P=0.997 | 0.01 (-0.03; 0.05) P=0.604 |
| Change baseline to 48 weeks | 2.14 (-2.74; 7.02) P=0.390 | -1.89 (-6.90; 3.12) P=0.460 | 0.01 (-0.04; 0.05) P=0.814 | -0.05 (-0.09; -0.01) P=0.027 |
| Difference between groups baseline | 2.72 (-4.71; 10.15) P=0.473 | | 0.01 (-0.04; 0.06) P=0.720 | |
| Difference between groups 24 weeks | 1.00 (-6.85; 8.84) P=0.803 | | 0.00 (-0.06; 0.06) P=0.998 | |
| Difference between groups 48 weeks | 6.75 (-1.81; 15.31) P=0.122 | | 0.06 (-0.00; 0.13) P=0.052 | |

**Supplemental table 1:** Overview QoL data (FACT-M and EQ-5D-5L) for melanoma patients included in the PROMelanoma study-intervention group vs. control group

**Supplemental table 2**: Overview QoL data (FACT-M and EQ-5D-5L) for melanoma patients included in the PROMelanoma study-grade 3 or 4 irAEs vs. no grade 3 or 4 irAEs

|  | FACT-M | | EQ-5D-5L | |
| --- | --- | --- | --- | --- |
|  | **Grade 3 or 4 irAEs** | **No grade 3 or 4 irAEs** | **Grade 3 or 4 irAEs** | **No grade 3 or 4 irAEs** |
|  | Coefficient (95% CI) P-value | Coefficient (95% CI) P-value | Coefficient (95% CI) P-value | Coefficient (95% CI) P-value |
| Change baseline to 24 weeks | -1.57 (-7.96; 4.82) P=0.629 | 1.40 (-2.13; 4.92) P=0.437 | -0.02 (-0.07; 0.04) P=0.573 | 0.01 (-0.02; 0.04) P=0.461 |
| Change baseline to 48 weeks | -3.53 (-11.05; 3.99) P=0.358 | 1.32 (-2.67; 5.30) P=0.517 | -0.05 (-0.12; 0.02) P=0.138 | -0.01 (-0.05; 0.02) P=0.481 |
| Difference between groups baseline | -2.09 (-10.71; 6.54) P=0.635 | | -0.02 (-0.08; 0.04) P=0.554 | |
| Difference between groups 24 weeks | -5.06 (-14.18; 4.06) P=0.277 | | -0.05 (-0.12; 0.02) P=0.174 | |
| Difference between groups 48 weeks | -6.93 (-17.08; 3.21) P=0.180 | | -0.06 (-0.14; 0.02) P=0.154 | |
